# High Caveolin-1 Expression in Tumor Stroma Is Associated with a Favourable Outcome in Prostate Cancer Patients Managed by Watchful Waiting

**DOI:** 10.1371/journal.pone.0164016

**Published:** 2016-10-20

**Authors:** Peter Hammarsten, Tove Dahl Scherdin, Christina Hägglöf, Pernilla Andersson, Pernilla Wikström, Pär Stattin, Lars Egevad, Torvald Granfors, Anders Bergh

**Affiliations:** 1 Department of Medical Biosciences, Pathology, Umeå University, Umeå, Sweden; 2 Department of Surgical and Perioperative Sciences, Urology and Andrology, Umeå University, Umeå, Sweden; 3 Department of Pathology and Cytology, Karolinska University Hospital, Stockholm, Sweden; 4 Department of Urology, Central Hospital, Västerås, Sweden; Medizinische Universitat Innsbruck, AUSTRIA

## Abstract

In the present study we have investigated whether Caveolin-1 expression in non-malignant and malignant prostate tissue is a potential prognostic marker for outcome in prostate cancer patients managed by watchful waiting. Caveolin-1 was measured in prostate tissues obtained through transurethral resection of the prostate from 395 patients diagnosed with prostate cancer. The majority of the patients (n = 298) were followed by watchful waiting after diagnosis. Tissue microarrays constructed from malignant and non-malignant prostate tissue were stained with an antibody against Caveolin-1. The staining pattern was scored and related to clinicopathologic parameters and outcome. Microdissection and qRT-PCR analysis of Cav-1 was done of the prostate stroma from non-malignant tissue and stroma from Gleason 3 and 4 tumors. Cav-1 RNA expression was highest in non-malignant tissue and decreased during cancer progression. High expression of Caveolin-1 in tumor stroma was associated with significantly longer cancer specific survival in prostate cancer patients. This association remained significant when Gleason score and local tumor stage were combined with Caveolin-1 in a Cox regression model. High stromal Caveolin-1 immunoreactivity in prostate tumors is associated with a favourable prognosis in prostate cancer patients managed by watchful waiting. Caveolin-1 could possibly become a useful prognostic marker for prostate cancer patients that are potential candidates for active surveillance.

## Introduction

Prostate cancer is the most common malignancy in Sweden and represents 32% of all cancer cases in Swedish men. Some of these tumors are indolent and can be left without treatment whereas others are highly aggressive and require early and effective treatment. For prediction of tumor aggressiveness Gleason scoring (GS) of tumor morphology is used [1]. More than 70% of the detected tumors are however graded as GS 6 or 7 and for these groups this does not predict outcome sufficiently well [2]. New prognostic markers are therefore needed.

Prognostic markers are generally searched for in the tumor epithelium but alterations in the prostate tumor stroma may also influence tumor growth and spread, and can thus be used to evaluate tumor aggressiveness. Changes in tumor stroma gene-expression, cellular composition, extracellular matrix, angiogenesis, hormone and growth factor receptors influence tumor progression, disease aggressiveness, treatment response and clinical outcome [3–10], but the mechanisms involved are largely unexplored. Stromal changes related to aggressive prostate cancer are decreased androgen [3] and transforming growth factor beta (TGF-beta) receptors [4–6], increased platelet derived growth factor receptor beta (PDGFR-beta) [7], increased hyaluronic acid [8], decreased density of mast cells [9] and increased density of macrophages and blood vessels [10, 11]. Together these changes form a so called reactive tumor stroma [12], and PDGFR-beta signalling promotes tumor growth and spreading [13].

Stromal and epithelial changes are also seen in the normal appearing prostate tissue surrounding tumors [3, 7–9, 11, 14–17]. Such changes could be of two partly overlapping mechanisms a) signs of “field cancerization”, that is discrete precancerous epithelial changes caused by the cancerogenic agent [18] or b) an adaptive response in previously normal epithelial or stroma cells to the presence of an already established tumor elsewhere in the organ (named TINT = Tumor Instructed/Indicating Normal Tissue [19]). The nature and magnitude of such changes are related to tumor grade and patient outcome, suggesting that changes in the tumor-bearing organ could be a novel source of prognostic markers [20].

The protein Caveolin-1 (Cav-1) is a major structural component of caveolae and acts as a scaffold within this domain. The protein is involved in a diversity of cellular processes including signal transduction, molecular transport and cell adhesion [21–23]. Cav-1 expression is high in terminally differentiated and quiescent cells, such as endothelial and smooth muscle cells [24]. Moreover, Cav-1 has been observed to be up- or down-regulated in various types of cancers [24]. In prostate tumor epithelium an up-regulation of Cav-1 has been reported and associated with high GS, extra-prostatic extension and lymph node involvement and with a poor outcome in patients treated with radical prostatectomy [25–28]. Further, a low stromal Cav-1 expression in prostate tumors is related to advanced tumor stage and metastatic disease and increased risk of PSA-relapse after radical prostatectomy [29, 30]. This suggests that high Cav-1 expression in prostate tumor stroma could perhaps be used as a marker for disease not needing treatment. We therefore examined if altered expression of Cav-1 in the tumor stroma and in the surrounding TINT is related to other functionally important changes in the tumor stroma, and if it can be used to predict outcome also in patients followed without treatment. For this purpose we used a historical cohort of prostate cancer patients diagnosed after transurethral resection of the prostate and managed by watchful waiting [1], to explore potential markers that could identify cases that can be safely left without treatment (such markers cannot be identified in patients that have been given curative treatments). This cohort has long follow-up and numerous of prognostic markers in the stroma have already been examined both in the tumor and in the surrounding TINT. The present findings show that high Cav-1 in tumor stroma is associated with a favourable outcome.

## Materials and Methods

### Ethics statement

All experiments were performed in accordance with relevant guidelines and regulations. The research ethical committee at Umeå university hospital (Regional Ethical Review Board), Umeå, Sweden has approved the handling of tissue samples and patient data in the present study. Tissue samples were registered as a case number and year in a database used for the analyses, with no names or personal identification number indicated. The paraffin material was collected according to Swedish regulations at a time when informed consent was not required. The research ethical committee at Umeå university hospital approved of the study and waived the need for consent. For the frozen material written informed consent was given and the research ethical committee at Umeå university hospital approved of the study.

### Patients

Between 1975 and 1991 tissue specimens were obtained from patients who underwent transurethral resection of the prostate (TURP) at the hospital in Västerås, Sweden, due to obstructive voiding problems, and subsequent histological analysis showed presence of prostate cancer. At that time serum prostate-specific antigen (PSA) was not yet used for diagnostics in Sweden. Tissue specimens were formalin-fixed, paraffin-embedded and re-graded according to the Gleason system [1]. Radionuclide bone scan was performed shortly after diagnosis for detection of metastases. Patients had not received any anti-cancer therapy prior to TURP. The study includes 395 patients of which 298 patients were followed by watchful waiting after TURP. At symptoms from metastases these patients received palliative treatment with androgen ablation and in a few cases radiation therapy or oestrogen therapy, according to therapy traditions in Sweden during that time period. Additionally 97 patients were also included in the study which hade been treated with palliative treatment immediately after diagnosis. The cause of death was assessed by evaluation of medical records. Cancer-specific and relative survival was almost similar, indicating a correct evaluation of the cause of death [31]. From the tissue specimens collected we constructed tissue microarrays (TMA) using a Beecher Instrument (Sun Prairie, WI, USA). The TMA:s contained 5–8 samples of tumour tissue from each patient representing both the primary and secondary Gleason grade (cores with a diameter of 0.6 mm), and 4 samples of non-malignant tissue from each patient. Each TMA contained samples from 8–10 patients.

We also retrieved frozen tumor tissue from 8 patients operated with radical prostatectomy, which were graded as Gleason score 7 (3+4; Gleason grade 3 and 4 being the most predominant and second most predominant patterns, respectively, in the tumor).

### Immunohistochemistry

Four micron thick paraffin sections were deparaffinated, rehydrated, treated with Diva antigen retrieval (BioCare, Concord, CA, USA) and microwave heated for 1 hour. Sections were then stained with a rabbit polyclonal anti-Cav-1 IgG (1:1000; N-20, directed against the N-terminal of human Cav-1, Santa Cruz Biotechnology, Santa Cruz, CA, USA) and visualized using Ventana DAB iView detection kit. For comparison, TMAs from 19 individuals were also stained with an antibody against the Cav-1 C-terminus (1:250; Epitomics, Burlingame, CA, USA) and visualized by Ventana Ultra view universal DAB detection kit. TMAs from 15 patients were double stained with antibodies against Cav-1 (Santa Cruz), visualized by Ultra view universal DAB detection kit, and anti-SMA (1:150; Actin (smooth muscle) Clone 1A4, Dako Denmark A/S, Glostrup, Denmark), visualized by Dako Envision K4018, and anti-Desmin (1:50; Desmin, Dako Denmark A/S, Glostrup; Denmark) visualized by Ventana Ultra view universal AP Red detection kit, respectively. From a subset of patients consecutive sections from the original tissue blocks were stained for Cav-1 as described above and for PDGFRβ as earlier described [7].

### Scoring of Cav-1 staining

**Fig 1:**
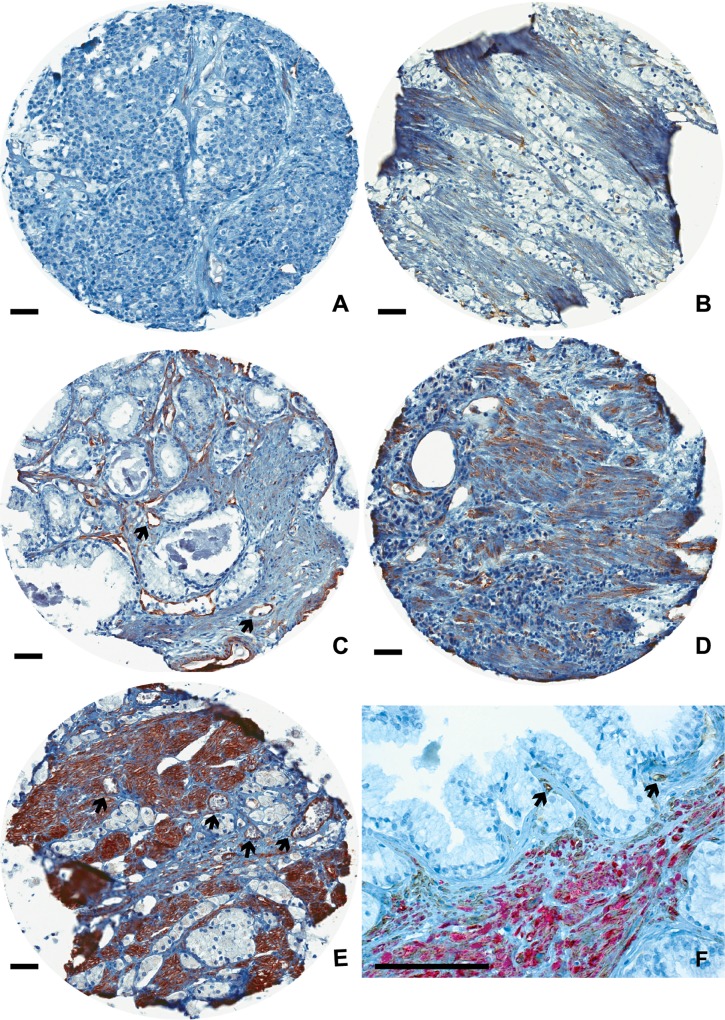
Prostate cancer stroma immunostained for Caveolin-1. Bars indicate 10 μm. The TMA core diameter is 0.6 mm and blood vessels marked by arrows. (**A**) No staining (score 0). (**B**) Weak, diffuse staining in less than 30% of stroma (score 1). **(C**) Weak, diffuse staining in more than 30% of stroma (score 2). (**D**) Moderate staining in more than 30% of stroma (score 3). (**E**) Strong staining in more than 30% of stroma (score 4). (**F**) Benign prostate tissue double stained for Caveolin-1 (brown) and Desmin (red). Most stroma cells were positive for both markers except blood vessels that were mainly Caveolin-1 positive.

The immunoreactivity for Cav-1 was evaluated without any knowledge of patient data and was performed by two different investigators (T.D.S., 395 patients and P.A., 47 patients), an intra-class correlation analysis using a mixed model and testing for consistency gave a Chronbach’s alpha [32] of 0.8. In order to quantify the Cav-1 immunostaining, a score combining staining intensity and distribution was used. Tumor stroma and non-malignant (normal tissue) stroma were quantified separately. Staining intensity and distribution for Cav-1 stroma was scored as 0 (no staining) (Fig 1A), 1 (weak, diffuse staining in less than 30% of stroma) (Fig 1B), 2 (weak, diffuse staining in more than 30% of stroma) (Fig 1C), 3 (moderate staining in more than 30% of stroma) (Fig 1D) and 4 (strong staining in more than 30% of stroma) (Fig 1E). The few cases with moderate and strong staining in less than 30% of stroma were scored as 2 and 3, respectively. The mean value of the scores for tumor stroma tissue and non-malignant stroma tissue, respectively, were used in the statistical analyses.

In a subset of our samples the immunoreactivity for Cav-1 tumor epithelium was evaluated without any knowledge of patient data and was performed by one investigator (P.H.). Scoring was performed in the same way as described above for Cav-1 stroma. Staining intensity and distribution for Cav-1 tumor epithelium was scored as 0 (no staining), 1 (weak, diffuse staining in less than 30% of tumor epithelium), 2 (weak, diffuse staining in more than 30% of tumor epithelium), 3 (moderate staining in more than 30% of tumor epithelium) and 4 (strong staining in more than 30% of tumor epithelium). The cases with moderate and strong staining in less than 30% of the epithelium were scored as 2 and 3, respectively. The mean value of the scores for tumor epithelium was used in the statistical analyses.

### Microdissection and qRT-PCR analysis of the prostate stroma

Fresh frozen prostate tissue sections from four different patients with Gleason score 7 (3+4) tumors were used for microdissection of stroma from non-malignant tissue, stroma within Gleason grade 3 and stroma within Gleason grade 4. Prior to microdissection, tissue sections were stained for 2 minutes with 50% hematoxylin followed by dehydration for 30 s in 70%, 95% and 100% ethanol respectively. Microdissection was performed using the PALM Laser-MicroBeam System. RNA was isolated with the PicoPure RNA Isolation Kit (Arcturus Engineering Inc.) in accordance with the manufacturer’s instructions. The total RNA isolated from these patients was converted to cDNA using SuperScript III reverse transcriptase (Life Technologies, Carlsbad, CA, USA) and Cav-1 expression was analysed with qRT-PCR with an ABI 7900 HT fast Real-Time PCR instrument (Applied Biosystems, Foster City, CA, USA) using power SYBR green (Life Technologies). The PCR reactions for Cav-1 were carried out with primers (forward: 50-CAAATGCCGTCAAAACTGTG-30, reverse: 50-CGACCCTAAACACCTCAACG-30). The PCR reactions for the internal control RPL13A were carried out with primers (forward: 50-GTACGCTGTGAAGGCATCAA-30, reverse: 50-GTTGGTGTTCATCCGCTTG-30).

### Cav-1 relation to prognostic markers in prostate cancer

Data on tumour size, local tumour stage, microvessel density, Ki-67 labelling index, metastasis, Gleason score, phosphorylated epidermal growth factor receptor (pEGFR), phosphorylated AKT (pAKT), hyaluronan, androgen receptor (AR), platelet-derived growth factor receptor beta (PDGFRβ) and angiogenesis have previously been quantified for these tumours [1, 3, 7, 8, 11, 14, 15, 33] and these data were related to Cav-1 levels.

### Statistics

Bivariate correlations were calculated using the Spearman’s rank correlation test. Correlations between nominal variables and continuous variables were analysed using the Kendall’s tau b correlation test. Data used in the correlation analysis was collected at the time of prostate cancer diagnosis. Patients included in survival analyses with Kaplan-Meier and Cox regression were followed by watchful waiting. The duration of event-free survival (EFS) is defined as the time from TURP until the date of prostate cancer death, death of other causes, or if no death occurred, until the date of last follow-up. Differences in outcome between groups were tested with the log-rank test. The prognostic relevance of Cav-1 immunoreactivity was examined by Cox regression analysis alone and together with GS. Means and medians are presented ± standard deviation (SD) and probability of event-free survival (P-EFS) is presented ± standard error (SE). The level of statistical significance was defined as *P* <0.05 (two-sided). Statistical analysis was performed using the SPSS 20.0.0 software for Os X (SPSS Inc., Chicago, IL, USA).

## Results

### Immunoreactivity for Cav-1

Immunoreactivity for Cav-1 was present in non-malignant (data not shown) and prostate tumor stroma (Fig 1). Most of the stroma in non-malignant prostate tissue was positive for Cav-1 but generally some cells were Cav-1 negative. The staining pattern resembled the Cav-1 staining in prostate tissue as reported in the Human Protein Atlas [34]. Blood vessels both in tumors and in the surrounding non-malignant prostate tissue, were positive for Cav-1 (in line with earlier observations) [29, 30] and served as an internal positive control (Fig 1). An additional Cav-1 antibody, against the C-terminus of the protein, was also used to explore antibody’s specificity. The staining pattern for this antibody was very similar to the antibody used for the whole material (data not shown). Occasionally Cav-1 positive cells were also found in prostate cancer epithelium, as earlier described [25–28]. However, when sections were stained with a less diluted Cav-1 antibody more epithelial staining occurred.

Cav-1 immunoreactivity in the tumor epithelium was significantly correlated to Cav-1 tumor stroma (r = -0.65, p<0,005, n = 40). Cav-1 in tumor epithelium in subgroups of patients with high (n = 20) respectively low (n = 20) Cav-1 in tumor stroma was significantly different (1.4 ± 0.60 vs. 2.3 ± 0.79, P < 0.001, respectively)

To characterize Cav-1 positive cells, a double staining with Cav-1 + Smooth muscle actin (SMA) and Cav-1 + Desmin were performed. Desmin is a marker for mature smooth muscle cells [3]. Most Desmin positive cells in the non-malignant prostate stroma were also Cav-1 positive (Fig 1). Prostate fibroblasts often located close to glands [3] were often Cav-1 negative (Fig 1).

In tumor stroma the amount of Desmin positive cells was low (as earlier described) [12] and cells positive for Cav-1 in tumor stroma were generally also Desmin positive. No Desmin positive Cav-1 negative cells were found. Elongated Cav-1 positive but Desmin negative cells (non muscle cells) were however observed and such areas were generally SMA positive suggesting that some myofibroblasts (cancer associated fibroblasts, CAFs) express Cav-1. The intensity and distribution of Cav-1 staining (both in the tumor and TINT stroma) did vary between patients and to some extent also between different tissue cores in the same individual.

### Caveolin-1 mRNA expression

**Fig 2:**
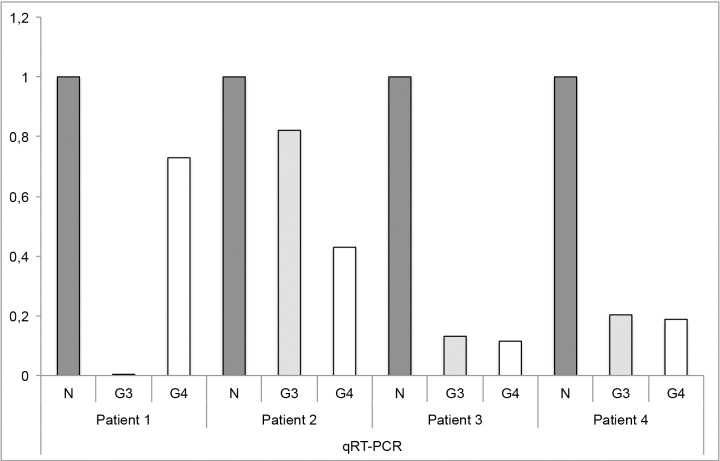
Tumor stromal Cav-1 expression. Cav-1 expression was analysed using micro dissection with qRT-PCR analysis. This was performed on non-malignant prostate stroma (N), stroma from a Gleason grade 3 tumor area (G3) and stroma from a Gleason Grade 4 area (G4) (all from the same patient). The tissue was derived from four different Gleason score 7 (3+4) tumors. Cav-1 expression was highest in normal prostate tissue and decreased with cancer progression.

The expression of Cav-1 was also analysed in micro-dissected stroma from normal prostate tissue, stroma from prostate cancer with Gleason grade 3 and stroma from Gleason grade 4 (4 patients were analysed with qRT-PCR). Cav-1 expression was highest in non-malignant tissue and decreased during cancer progression (Fig 2).

### Correlation analysis

Cav-1 immunoreactivity in prostate tumor stroma was weakly inversely correlated with GS, metastasis, tumor size, local tumor stage, tumor cell proliferation, tumor stroma hyaluronan, tumor vascular density and tumor epithelial pEGFR expression, and weakly positively correlated with non-malignant stroma Cav-1 immunoreactivity and tumor stroma androgen receptor expression (Table 1). Tumor stroma Cav-1 was not correlated to PDGFRβ in tumor stroma (see below).

**Table 1:** Bivariate correlations.

		Tumor stroma Cav-1
Non-malignant stroma Cav-1*	r	0.30^†^
	n	344
Gleason score*	r	-0.42^†^
	n	373
Local tumour stage*	r	-0.34^†^
	n	366
Metastases^‡^	r	-0.28^†^
	n	292
Tumor size*	r	-0.42^†^
	n	373
Tumor cell proliferation*	r	-0.25^†^
(Ki-67 labelling index)	n	367
Tumor stroma hyaluronan*	r	-0.27^†^
	n	369
Tumor vascular density*	r	-0.21^†^
(vWf-stained vessels)	n	363
Tumor epithelial pEGFR*	r	-0.23^†^
	n	284
Tumor stroma AR*	r	0.33^†^
	n	365
Tumor epithelial pAKT *	r	-0.29^†^
	n	275
Tumor stroma PDGFR β*	r	-0.069
	n	280

Notes: Data used in the correlation analysis were collected at the time of prostate cancer diagnosis.

Abbreviations: pEGFR, phosphorylated epidermal growth factor

vWF, von Wilebrand factor; AR, androgen receptor; pAKT, phosphorylated AKT.

*Spearman’s rank correlation test.

†Correlation is significant at the <0.005 level (2-tailed).

‡Kendall’s tau b correlation test.

### Cut-offs used in the survival analysis

**Fig 3:**
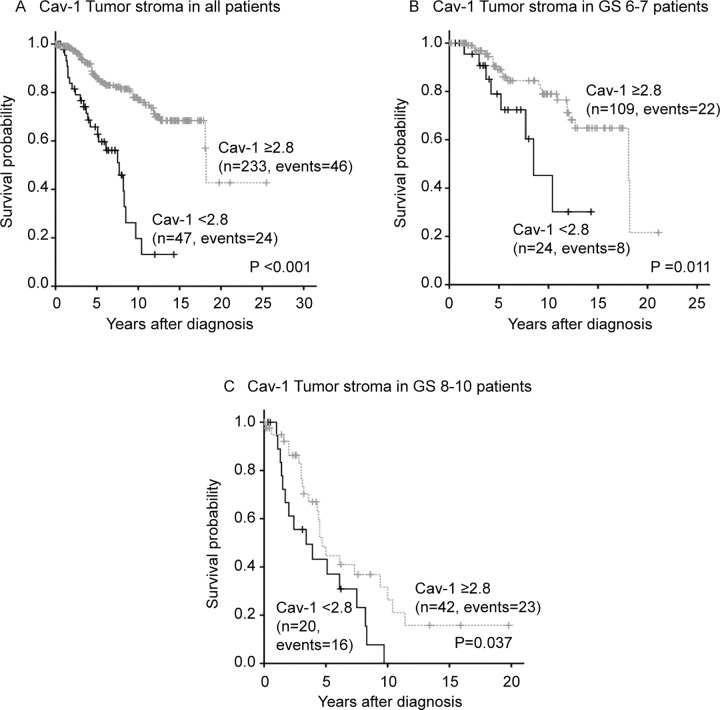
Kaplan-Meier survival curves. Patients divided into two groups depending on immunoreactivity of tumor stroma Caveolin-1 (Cav-1) in all patients (**A**), patients with GS 6–7 (**B**) and patients with GS 8–10 (**C**). Dashed line, high tumor Cav-1 (≥2.8, quartiles 2, 3,4); solid line, low tumor Cav-1 (<2.8, 1^st^ quartile).

The cut-off used in survival analysis was the first quartile (= 2.8) for tumor stromal Cav-1 staining, i.e. high tumor stroma Cav-1 immunoreactivity was ≥2.8. This cut-off was used in Kaplan-Meier and COX analysis for the whole watchful waiting material (n = 280; Fig 3). These survival analyses showed that high Cav-1 immunoreactivity in prostate tumor stroma was associated with a significantly longer cancer specific survival (Fig 3A). Further analyses of the whole watchful waiting material showed that the cut-off could also be set to 3, obtaining similar results in Kaplan-Meier (results not shown). Thus, demonstrating that patients with moderate (score 3) or high Cav-1 staining (score 4) in more than 30% of the stroma had a good prognosis also in the absence of curative treatment.

### Tumor stroma Cav-1 immunoreactivity and clinical outcome

Patients with low tumor Cav-1 had significantly shorter cancer specific survival than patients with high tumor Cav-1 (10-year probability of event-free survival, P-EFS, was 20 ± 9.5% and 77 ± 3.5% in the two groups; Fig 3A). In addition, low tumor Cav-1 in patients with GS 6 + 7 (10-year P-EFS 45 ± 17%) had significantly shorter cancer specific survival than those with high tumor Cav-1 in this subgroup (10-year P-EFS 79 ± 5%) (Fig 3B). Cav-1 was not associated with prognosis in Gleason 6 or Gleason 7 tumors when analysed separately, possibly because the number of patients in each group was too low). A difference in survival between groups with high and low Cav-1 was also seen in patients with GS 8–10 (Fig 3C). Low tumor Cav-1 was associated with an increased relative risk for prostate cancer specific death in a univariate Cox regression analysis (Table 2). In multivariate Cox regression analysis including the known prognostic marker GS and local tumour stage, low tumor Cav-1 was significantly associated with poor prognosis and gave additional prognostic information (Table 2).

**Table 2:** Cox regression for tumor stroma Cav-1 of patients followed by watchful waiting.

Variable		n	RR	*P*-value	95% CI
*Univariate analysis*					
Gleason score*	4–5	91	1^†^		
	6–7	150	25.0	0.002	3.4–182.9
	8–10	63	128.7	<0.001	17.6–939.5
Local tumor stage*	T1a+T1b	189	1^†^	<0.001	
	T2	74	4.0	<0.001	2.3–7.0
	T3	35	9.8	<0.001	5.4–17.8
	T4	3	11.6	0.02	1.5–88.1
Tumor stroma Cav-1*	≥2.8	233	1^†^		
	<2.8	47	4.5	<0.001	2.7–7.5
*Multivariate analysis*					
Gleason score*	4–5	84	1^†^		
	6–7	133	19.8	0.003	2.7–145.8
	8–10	60	67.4	<0.001	8.8–517.6
Tumor stroma Cav-1*	≥2.8	230	1^†^		
	<2.8	47	2.1	0.007	1.2–3.7
Local tumor stage*	T1a+T1b	171	1^†^		
	T2	73	1.6	0.14	0.9–3.0
	T3	30	1.7	0.18	0.8–3.9
	T4	3	5.0	0.12	0.7–38.7

*Cox regression analysis using Gleason score, local tumor stage and tumor stroma Cav-1 as categorical variables.

^†^Reference value.

Abbreviations: RR, relative risk; CI, confidence interval.

### Tumor stromal Cav-1 and PDGFRβ immunoreactivity and clinical outcome

**Fig 4:**
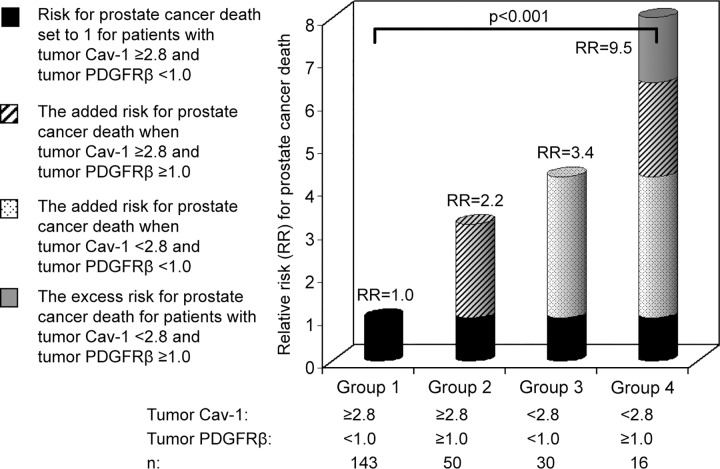
Tumor stroma Cav-1 and PDGFR interaction. The diagram shows the interaction, with regard to outcome, between tumor stroma Cav-1 and tumor stroma PDGFR beta immunoreactivity. It also illustrates the excess risk/interaction effect of low tumor stroma Cav-1 and high tumor stroma PDGFR beta in patients followed by watchful waiting. The groups show the relative risk (RR) for prostate cancer death.

Since Cav-1 and PDGFRβ in tumor stroma appears to be unrelated (Table 1) but both add prognostic information about prostate cancer clinical outcome [7] a combined variable between the two was constructed and analysed by Cox regression. The few patients (7%) with low tumor Cav-1 and high tumor PDGFRβ had an approximately 3-fold excess risk of prostate cancer death compared to the added separate relative risks for patients identified as having low tumor Cav-1, or high tumor PDGFRβ respectively (interaction analysis *P* <0.001) (Fig 4).

**Fig 5:**
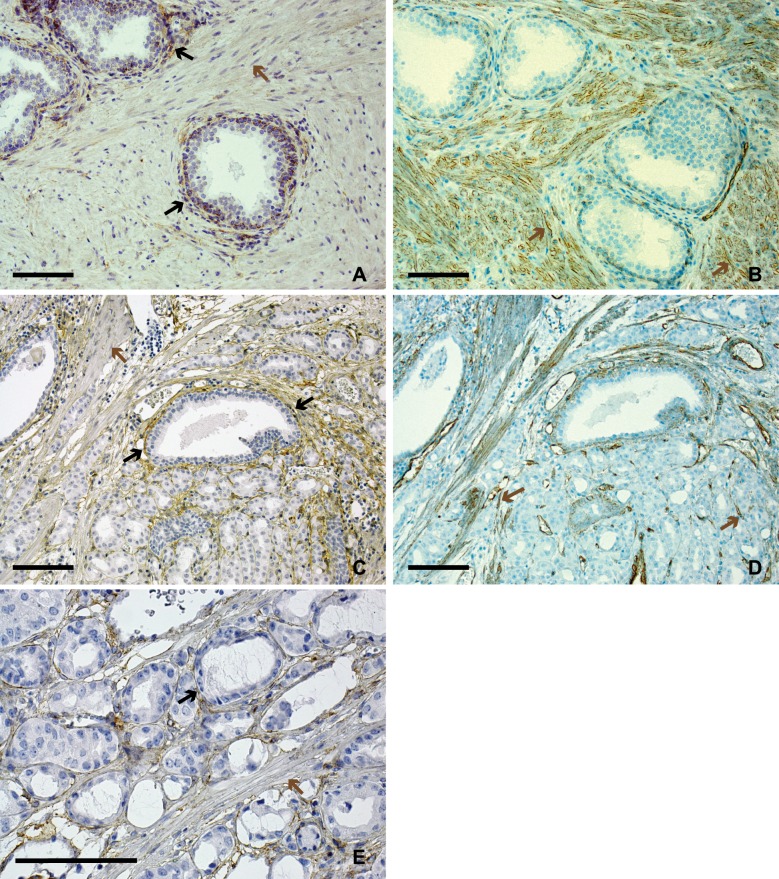
Prostate tissue and tumor immunostained for PDGFRβ. Consecutive sections of benign prostate tissue (A, B) and tumor tissue (C, D, E) stained for PDGFRβ (A, C, E) and Cav-1 (B, D). Bars indicate 10 μm. Elongated fibroblast-like cells (marked by black arrows) lying close to benign or malignant glands are generally PDGFRβ positive and Cav-1 negative. Elongated smooth muscle like cells (marked by brown arrows), both in benign and tumor tissue, are generally PDGFRβ negative and Cav-1 positive. Cells in blood vessel walls may express both PDGFRβ and Cav-1.

To explore this further, consecutive sections were immunostained for Cav-1 and PDGFRβ. In benign prostate tissue PDGFRβ staining was observed in the fibroblast like cells situated directly adjacent to glands, whereas the smooth muscle cells normally situated in the more central parts of the stroma were negative (Fig 5A). Cav-1 staining in benign prostate tissue showed the opposite pattern with strong staining in smooth muscle cells but limited in fibroblast-like cells adjacent to glands (Fig 5B). In tumors PDGFRβ staining was detected in the fibroblast like cells lying close to neoplastic cells (Fig 5C). These cells were generally Cav-1 negative and Cav-1 positivity was instead detected in sheets of remaining smooth muscle cells (Fig 5D).

## Discussion

The principal finding in this study is that a decreased immunoreactivity of Cav-1 in the prostate tumor stroma was correlated to a poor outcome in prostate cancer patients managed by watchful waiting. In line with previous studies [29, 30] we also found that Cav-1 in tumor stroma was weakly inversely correlated to tumor size, tumor stage, Gleason score and metastasis at diagnosis, and to PSA-relapse in surgically treated men. Prostate cancer is thus similar to breast cancer where decreased Cav-1 expression in the tumor stroma is associated with aggressive disease [35]. In the present study tumor stroma Cav-1 added prognostic information in addition to current clinical prognostic markers such as GS and local tumor stage. Cav-1 in tumor stroma also gave prognostic information for patients with GS 6–7 graded tumors. A group that constitutes more than 70% of the localized tumors and that have a largely unpredictable outcome [2]. Unfortunately, the number of patients with GS 7 tumors was too low to analyse the role of Cav-1 in this important subgroup. Importantly patients with high Cav-1 expression in the tumor stroma had an excellent outcome also in the absence of active treatment. Such men could, when the current findings have been validated in other studies, particularly in diagnostic needle biopsies, be suitable candidates for active monitoring instead of an early curative treatment.

Cav-1 staining was present in elongated smooth muscle and fibroblast-like cells in the stromal compartment as earlier observed [29, 34]. In the epithelial compartment immunostaining was occasionally seen, but apparently not in the same extent as previously reported by for example DiVizio et al [29]. The reason to this discrepancy is probably methodological as higher antibody concentrations increased epithelial staining also in our study, and high epithelial staining was in our study correlated to low stroma staining. Cav-1 was in normal prostate tissue predominantly expressed in Desmin and SMA positive cells, which is expressed in smooth muscle cells. In prostate cancer stroma smooth muscle cells are reduced and the magnitude of this predicts biochemical recurrence in prostate cancer patients [12]. The reduction of Cav-1 in tumor stroma could therefore be related to loss of differentiated smooth muscle cells. This can probably not be the only explanation as some SMA positive cells in the tumor stroma, presumably cancer associated fibroblasts (CAFs), were Cav-1 positive whereas others were negative.

Cav-1-expression in tumor stroma was weakly correlated to other prognostic markers previously found in this patient material, such as decreased AR [3], increased hyaluronan [8] and increased vascular density [11] in tumor stroma, and increased phosphorylated EGF-receptors, phosphorylated AKT and cell proliferation in tumor epithelial cells [14, 15]. Interestingly, Cav-1 was not associated to levels of PDGFR beta staining in tumor stroma, a factor previously associated with a poor prognosis [7]. Patients with low stromal Cav-1 and high stromal PDGFR beta (seen only in 7% of the patients in this material) was found to have an approximately 3-fold excess risk of prostate cancer death compared to the other patient groups suggesting that a combination of these two types of stroma has a synergistic effect on tumor aggressiveness. PDGFRβ is normally expressed in the fibroblasts lying closest to prostate glands. Possibly these cells, in response to signals from neoplastic cells, are turned into CAFs expressing high levels of PDGFRβ. Cav-1 is expressed in smooth muscle cells that dedifferentiate and often loose their Cav-1 expression during tumor progression. The observation that Cav-1 and PDGFRβ expression were non-correlated suggest that these two processes could be differently regulated. It therefore appears that there could be two separate ways to acquire a stroma that is associated with poor prognosis, one related to smooth muscle dedifferentiation (decreased Cav-1) and another related to increased number of peri-glandular derived CAFs (high PDGFRβ). Further studies are however needed to test this hypothesis.

A weakness of this study is the scoring system of the Cav-1 immunoreactivity. This scoring is, although reproducible, subjective and the separation between high and low score (between none-weak or moderate-strong) is probably not ideal. Another potential weakness is that the current study is based on TURP derived tissue from men with voiding problems. This tissue may be different from tissue obtained through peripheral zone diagnostic needle biopsies. This may however be of limited importance for the current results, as aggressive peripheral zone cancers in radical prostatectomy specimen appear to have the same alteration in Cav-1 as in our study [30].

Although this and previous studies [30] suggests that decreased stromal Cav-1 is associated with poor prostate cancer prognosis several important questions remain unanswered. What are the mechanisms behind the decrease? Is the decrease in Cav-1, caused by oxygen radicals from the tumor epithelium, mechanistically involved in creating a stroma with low Cav-1 CAFs that promote tumor cell aggressiveness by providing metabolites, as suggested for breast cancer by Lissanti and coworkers [36], or is it a marker for other changes more directly associated with tumor aggressiveness such as tumor hypoxia (hypoxia is reported to decrease Cav-1 in CAFs, 38)? Silencing of Cav-1 in prostate stroma cells induced a stroma phenotype effective in stimulating local androgen synthesis, angiogenesis, invasion and metastasis [30], suggesting that low Cav-1 expression in tumor stroma directly supports tumor growth. Alterations in tumor stroma are generally considered to be the result of paracrine and endocrine signalling from tumor epithelial cells to adjacent and more remote tissues [37, 38] suggesting that the decrease in Cav-1 could be a response to signals from the tumor epithelium specific for smooth muscle cells as endothelial Cav-1 appeared unaffected. As the various changes measured in the tumor stroma in this set of patients were only weakly correlated, or not correlated at all, it is not unlikely that there could be several signals from the tumor epithelium affecting different subsets of stroma cells.

Recent studies suggest that several factors altered in the stroma of prostate tumors, also are altered in the stroma of the surrounding non-malignant tumor-containing organ (the TINT,[20]). In most cases the alterations in tumor stroma and TINT follow each other [28]. Similarly Cav-1 expression in the surrounding non-malignant stroma was correlated to Cav-1 immunoreactivity in the tumor stroma and to other tumor characteristics, but the correlations were rather weak and Cav-1 immunoreactivity in non-malignant stroma was not related to outcome.
